# Enhancement of Biological Properties of Blackcurrants by Lactic Acid Fermentation and Incorporation into Yogurt: A Review

**DOI:** 10.3390/antiox9121194

**Published:** 2020-11-27

**Authors:** Rebecca Kowalski, Erika Gustafson, Matthew Carroll, Elvira Gonzalez de Mejia

**Affiliations:** Department of Food Science and Human Nutrition, University of Illinois, Urbana, IL 61856, USA; rkowals2@illinois.edu (R.K.); erikag3@illinois.edu (E.G.); mjc13@illinois.edu (M.C.)

**Keywords:** antioxidant capacity, blackcurrants, lactic acid fermentation, health benefits, metabolic health, neurological disease, *Ribes nigrum*, yogurt

## Abstract

Blackcurrants (BC) and yogurt are known to possess several health benefits. The objective of this review was to compile the latest information on the effect of lactic acid fermentation on BC and their incorporation into yogurt, including the impact of this combination on chemical composition, sensory aspects, and health attributes of the blend. Google Scholar, Scopus, and PubMed were used to research the most recent literature on BC juice, the whole BC berry, and yogurt. Health benefits were assessed from human and animal studies within the last 5 years. The results suggest that BC have several health promoting compounds that ameliorate some neurological disorders and improve exercise recovery. Yogurt contains compounds that can be used to manage diseases such as type 2 diabetes (T2D) and irritable bowel disease (IBD). Fermenting BC with lactic acid bacteria (LAB) and its incorporation into yogurt products increases the polyphenol and antioxidant capacity of BC, creating a blend of prebiotics and probiotics compounds with enhanced benefits. More research is needed in the area of lactic acid fermentation of berries in general, especially BC.

## 1. Introduction

**Figure 1:**
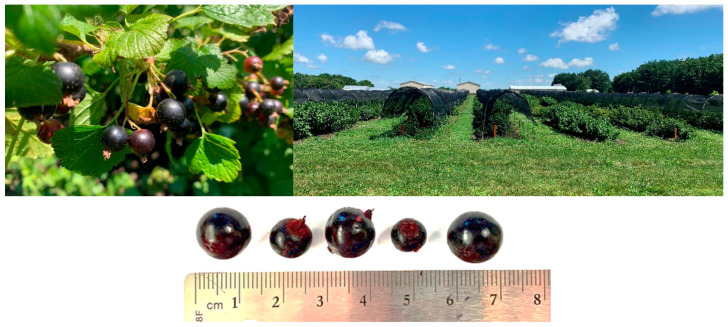
Blackcurrants grown in Urbana, Illinois by the Department of Crop Sciences, University of Illinois.

Blackcurrants (BC) (*Ribes nigrum*) (Figure 1) are small dark berries that originated in Northern Asia and Central and Eastern Europe [1]. This specie belongs to the genus *Ribes,* and family *Grossulariiaceae* [2]. Consumption of BC in the United States (U.S.) has been historically not very common because its cultivation was for some time legally prohibited. BC is a host for the fungus *Cronartium ribicola* that causes white pine blister rust on trees. The infection by *C. ribicola* in white pine can lead to girdling cankers and tree death. Due to this deleterious effect to trees, BC was banned and removed in the U.S. in the early 1900s [3,4]. Efforts to prevent infection by *C. ribicola* have been made. For example, the genetic development of BC resistant to *C. ribicola* [5,6]. Now that legislation to prevent the growth of BC in North America has been repealed, acreage of BC has increased [7].

Yogurt, on the other hand, is a food dating back to ancient times, and it is difficult to pinpoint the exact year of its discovery. It is believed that milk products were consumed by humans around 10,000–5000 BC when milk-producing animals were domesticated. Around this time herdsmen in the Middle East discovered that their bags made of intestines caused the milk to curdle and sour, which allowed it to be preserved for longer. Along with the Middle East, Indian Ayurvedic scripts from around 6000 BC referred to the benefits of consuming fermented milk products [8].

The popularity of yogurt has grown largely due the introduction of fruit-flavored products, since fruit allows for versatility in taste, color, and texture. Fruit also contains fiber, flavonoids, and other health promoting compounds that can protect against certain diseases [9].

The objective of this review was to compile the latest information on the effect of lactic acid fermentation on BC and its incorporation into yogurt, including the impact of this combination on chemical composition, sensory aspects, and health attributes.

## 2. Blackcurrants

According to the U.S. Department of Agriculture (USDA), the proximal composition of raw European BC is 81.96% water, 1.40% protein, 0.41% fat, 15.38% carbohydrates, and the remaining percentage constitutes are vitamins and minerals [10]. The sugar content, on average, is around 9% in the form of glucose (45%), fructose (40%), and sucrose (15%). The acid content is approximately 5% of fresh weight consisting of citric (88%) and malic acids (12%) [11].

### 2.1. Blackcurrants Chemical Characterization

BC contain a wide variety of bioactive compounds. One study has reported 63 chemical compounds identified by ultra-performance liquid chromatography-diode array detector electrospray-ionization tandem mass spectrometry (UPLC-DAD-ESI-MS) (Table 1). Authors found 15 anthocyanins (ANC), 19 flavanols, 14 phenolic acid derivatives, 6 sugars, 4 flavan-3-ols, and 4 organic acids [12]. ANC were the most dominant group of polyphenols, being the highest cyanidin and delphinidin derivatives, especially delphinidin-3-*O* rutinoside [12,13]. Furthermore, myricetin glycosides were the most common flavanols, and coumaric acid the highest phenolic acid. However, some cultivars have shown to contain more caffeic acid than coumaric acid. For organic acids, citric acid was the most prevalent and for sugars, fructose and glucose. Variation in content of phenolic compounds depended on factors such as type of cultivar, growing temperature, fruit ripening, and growing location [12,14,15,16,17].

Compared to other common berries, such as strawberries, blueberries, blackberries, and raspberries, BC contain higher levels of calcium, iron, magnesium, phosphorus, and especially potassium and ascorbic acid [18]. The antioxidant activity of BC, blueberries, raspberries, red currants, and cranberries have been measured using the ferric reducing antioxidant power (FRAP) assay. By this method, BC had the most antioxidant activity. In addition, HPLC analysis showed that BC contained the highest quantity of vitamin C and ANC (2328 and 5521 nmol/g of fresh weight, respectively), reflecting its high antioxidant capacity [19] (Table 2). In addition, BC contain a high amount of pectin (0.9–1.5%), which contributes to the viscosity of the berry that differs greatly from other berries [20]. Rheological properties depended on Brix°, pectin content, and the temperature at which the data was obtained [21].

On the other hand, orosensory properties of BC are affected by other important compounds. For example, kaempferol-3-*O*-(6″-malonyl) glucoside, myricetin-3-*O*-galactoside, and an unknown kaempferol glycoside, contribute to its astringency [22]. Furthermore, proanthocyanidins (PAC) in BC, also referred to as condensed tannins, are linked to astringent and bitter sensory properties. It is hypothesized that this phenomenon is partially due to polymeric tannins binding to salivary proteins in the mouth [23]. BC juiced with the aid of enzymes like pectinases are more astringent and bitter than when juiced without enzymes. This is because enzyme-aided juices contain more phenolic compounds (4–10 fold), a slightly higher pH (~0–0.2 increase), and a lower sugar/acid ratio (~0.6–1.1 increase). It is believed that the astringency of BC may be masked by pectins, and when these are broken down by pectinase, several compounds are released that affect the sensory attributes of the berry [24]. It is important to note that the liking of certain orosensory properties by consumers are greatly influenced by age, gender, and previous consumption of BC [25].

### 2.2. Health Benefits of Blackcurrants

**Figure 2:**
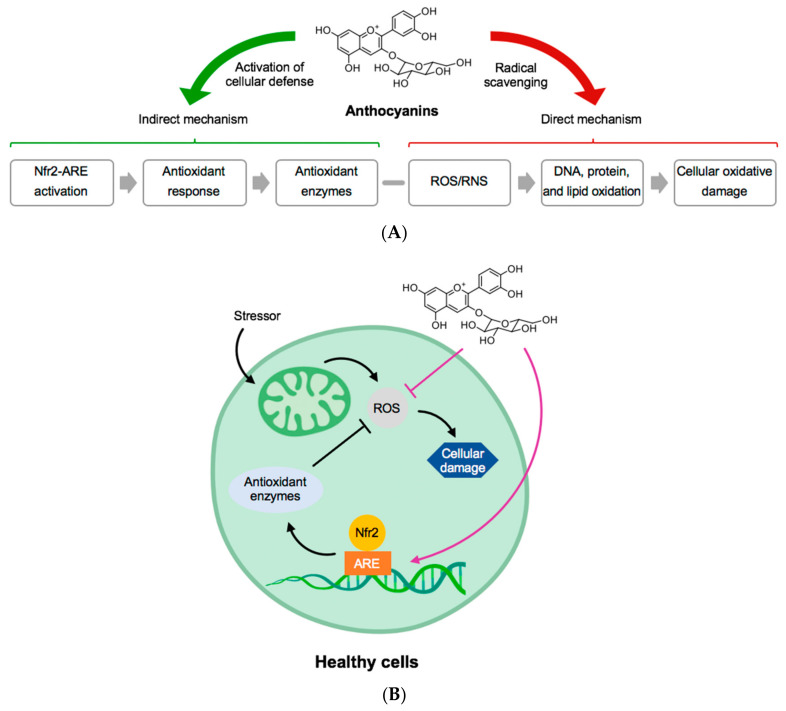
(**A**) General structure of anthocyanins and their mechanism of direct and indirect activation of antioxidant protection. (**B**) In a healthy cell, an example of how anthocyanins can block reactive oxygen species (ROS) and protect the mechanism for production of antioxidant enzymes. Abbreviations: ARE, Antioxidant response element; Nfr2, NF-E2-realted factor-2; RNS, reactive nitrogen species. Adapted from [26].

ANC and other phenolic compounds in BC can be linked to specific health benefits for humans such as ameliorating neurological disorders and improvement of exercise recovery. ANC are multimechanistic antioxidants that can control oxidative stress in healthy cells as presented in Figure 2 [26].

#### 2.2.1. Blackcurrants and Neurological Effects

**Figure 3:**
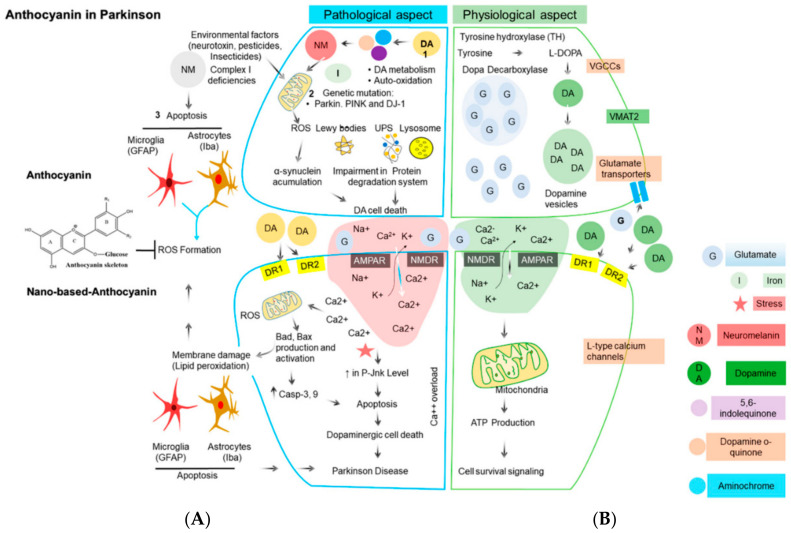
Major sources of oxidative stress in Parkinson’s disease and the corresponding antioxidant defense mechanism in dopaminergic neurons. (**A**) In a pathological state, reactive oxygen species (ROS) act through a series of mechanisms resulting in cell damage and lipid peroxidation, which causes Parkinson’s disease. When anthocyanins are involved, they block ROS. (**B**) In a physiological state, there is an absence of ROS, which promotes cell growth without lipid peroxidation and a healthy cell is seen. The arrows represent an uninterrupted pathway. The T represents a blocked pathway. Abbreviations: UPS, ubiquitin proteasome system; α-syn, α-synuclein; AMPA, α-amino-3-hydroxy-5-methyl-4-isoxazolepropionate; NMDA, *N*-methyl-d-aspartate; DR1 and DR2 (dopamine receptor 1 and 2); DA, dopamine, DJ-1, Protein deglycase [31]. Licensee MDPI, Basel, Switzerland. This article is an open access article distributed under the terms and conditions of the Creative Commons Attribution (CC BY) license (http://creativecommons.org/licenses/by/4.0/).

Parkinson’s disease (PD) is the second most common neurodegenerative condition. It is diagnosed based on symptoms such as slowness in movement (bradykinesia), tremor, rigidity, and difficulty with balance [27]. Disease progression occurs starting with resistance to insulin-like growth factor-1 (IGF-1) when circulating IGF-1 increases but function in neuronal survival and brain function decreases [28]. Cyclic glycine-proline (cGP), a metabolite of IGF-1 has been used to represent the bioavailability to IGF-1. Studies showed a significant increase in cGP (from 7.27 ± 0.67 ng/mL to 12.12 ± 0.94 ng/mL) with increased consumption of ANC in BC (300 mg twice daily for 4 weeks) (*p* < 0.01) and there was a strong correlation between the concentration of cGP in cerebral spinal fluid (CSF) and plasma (*p* = 0.01) [29]. This evidence showed that ANC in BC have the potential to alleviate neurological conditions when there is IGF-1 deficiency such as in PD. However, more evidence is needed because of the low number of participants in this study (11) and since evaluating IGF-1 function from cGP and cGP/IFG ratio in clinical research is relatively new [29]. BC contain both ANC and PAC [12], based on this consideration, a study on the compound rotenone showed that BC may have potential to lower the risk of PD. A patient with PD has a loss of dopaminergic neurons. Rotenone, a PD-related neurotoxin, can increase PD risk when a person is exposed to this compound for a long time in the environment. Therefore, berry extracts rich in ANC and PAC have been tested for their ability to alleviate rotenone neurotoxicity. Rotenone neurotoxicity reduces tyrosine hydroxylase (TH+) neurons by approximately 55% on average, but in the presence of BC extract at concentrations of 0.001 and 1.0 μg/mL, rotenone neurotoxicity reduced TH+ neurons by 22%. Not all ANC are neuroprotective and multiple ANC may be required to lower the risk of PD disease progression. The available data suggest that BC extracts followed alternate unknown mechanisms for suppressing rotenone neurotoxicity [30]. Figure 3 shows a representation of how ANC might protect against sources of oxidative stress in Parkinson’s disease [31].

Monoamine oxidase (MAO)-B inhibitors have been used to alleviate neurodegenerative symptoms associated with PD, and MAO-A inhibitors are utilized in the treatment of many mood disorders, such as depression. Supplementation with BC juice (142 mL obtained by a cold-press yielded from approximately 150 g of fresh ‘Blackadder’ cultivar) to a young healthy adult cohort inhibited MAO-A and MAO-B and induced a positive change in behavior. After supplementation with BC juice (525 ± 5 mg of polyphenols per 60 kg of bodyweight) from an anthocyanin-enriched BC extract (1.66 g of Just the Berries, Palmerston North, New Zealand, DelCyan™), there was a significant increase in attention [32,33]. Similar results were seen when both treatment groups were provided with 7.7 mg of ANC/kg (juice) and 8.0 ANC mg/kg (DelCyan). BC juice (4.8 mg/kg body weight) inhibited MAO-B activity and improved neurotransmission. There was a 90% (*p* < 0.001) reduction in platelet MAO-B activity after consumption of a BC drink 1 h before a low impact walking exercise compared to consumption of a placebo drink [34]. The fact that BC can improve neurotransmission and cognition by inhibiting MAO activity describes further the potential of BC to prevent neurological disorders.

BC have the potential to inhibit MAO-A activity and be useful in the treatment of mood disorders [32]. BC juice (142 mL) significantly reduced plasma normetadrenaline concentration by 60% and increased 3,4-dihydroxyphenylglycol (DHPG) concentration by ~36% in human subjects. These changes in DHPG and normetadrenaline concentration could be due to inhibition of peripheral MAO-A enzyme [32]. Studies have also shown that BC juice consumption (500 mg of polyphenols) can lower anxiety, allow for greater alertness, and lowers fatigue [35].

In summary, polyphenols in BC, such as ANC, have been shown to reduce neurotoxicity, benefit neurotransmission, and improve cognition, mood, and feeling, all of which would be beneficial to prevent or reduce the risk of neurological diseases such as Parkinson’s.

#### 2.2.2. Blackcurrants and Improving Exercise Recovery and Athletic Performance

The relation between BC and exercise have also been studied. Healthy participants who consumed BC juice (4.8 mg of polyphenols/kg bodyweight) 1 h prior to exercise reported a lower exertion score than those who were in the control group [34]. In addition, consumption of 1.6 or 3.2 mg/kg of BC extract (BCE) 1 h prior to exercise allowed enhanced recovery from exercise-induced oxidative stress compared to the placebo and 0.8 mg/kg of BCE [36]. After consumption of BCE and 30 min of rowing exercise by human subjects, there was a significant reduction in malondialdehyde, a lipid oxidation product (*p* < 0.05). In addition, anti-inflammatory properties were observed, both of which can lead to enhanced exercise recovery [37].

Positive benefits of BC during endurance exercise were observed in male cyclists randomly assigned to consuming different amounts of BCE (300, 600, or 900 mg per day for 7 days) on cardiovascular function. The results showed that cardiac output and stroke volume increased, and peripheral resistance decreased compared to the control [38]. Another study came to the same conclusion after analyzing the performance of cyclists. It was found that after consuming a BC powder (6 g/day), cycling intensity increased by 6% [39]. Cook et al. [40] also showed an improvement in cycling performance by 2.4% after BCE consumption (300 mg/day with 105 mg of ANC) and found an increase in fat oxidation by 27%. Delphinidin in BCE relaxed blood vessels; therefore, increased blood flow by increasing nitric oxide (NO) from the increase in calcium ion concentration in endothelial cells and reducing the breakdown of NO free radicals [40]. High-intensity intermittent runners improved performance and recovery after consuming BCE (300 mg/day with 105 mg ANC). They were able to run greater distances (10.6%) and had a larger change in blood lactate during recovery. Polyphenols had an effect on vascular function that allowed enhanced blood flow and improvements in athletic performance and exercise recovery [41]. In addition, BC extract supplementation (600 mg/day with 210 mg ANC) has also shown to improve climbing performance by improving total climbing time (+23%) compared to the placebo (−11%). This may be due to enhanced blood flow; however, supplementation did not prevent fatigue [42].

In summary, consumption of BCE has potential to lower exertion and improve cardiovascular function during exercise, improving recovery afterward. It is important to comprehend the mechanisms of actions for the ANC and other compounds in BC in relation to athletic performance and exercise recovery, highlighting the need of more clinical investigations in this field [43].

#### 2.2.3. Other Health Benefits of Blackcurrants

BC also have other health benefits related to diabetes, endothelial function, and inflammation. Table 3 shows the most recent studies on consumption of BC and their effect on various health indicators in humans. After subjects consumed a BC extract drink containing 600 mg of total ANC before a high-carbohydrate meal, plasma glucose, plasma insulin, plasma glucose-dependent insulinotropic polypeptide (GIP), and plasma glucagon-like peptide 1 (GLP-1) concentrations became significantly reduced compared to a control. The polyphenols inhibited intestinal glucose transporters and digestive enzymes to control glycemic response [44]. This evidence suggests the potential benefit of BC on diabetes.

To test the effect of BC on endothelial function, one study looked at smokers compared to nonsmokers. When young, healthy male smokers consumed BC ANC (50 mg), acute endothelial dysfunction was reduced within 2 h after taking the supplement. Instead of flow-mediation dilation (FMD) decreasing from 9.5% to 8.2% for the control group 1 h after smoking a cigarette, in the BC ANC supplemented group, the FMD was reduced to a lesser extent (9.5% to 8.7%, 1 h after smoking). In addition, after BC ANC supplementation, the FMD 2 h after smoking was closer to presmoking FMD in comparison to the control (from 9.5% to 8.9% for the control, and from 9.5% to 9.3% for the BC ANC supplemented group) [45]. The effect of BC juice on endothelium-dependent relaxation was tested using porcine coronary arteries. A positive correlation was found between relaxation amplitude and total ANC content of the BC juice [46]. Human subjects who took two capsules (300 mg capsule with 35% BCE) of a New Zealand BCE showed a decreased carotid-femoral pulse-wave velocity and central blood pressure compared to the placebo and baseline [47]. These studies showed the potential of BC juice to promote vascular protection and be beneficial for cardiovascular health.

Inflammation occurs as a response to an irritant in the body such as in the case of asthma. C-C motif chemokine ligand 11 (CCL11) is an important target for allergic airway therapy since it regulates the recruitment of eosinophils to the inflammation site. It was found in mice that BC ANC (10 mg/kg of total ANC of a commercially available ANC-rich New Zealand “Ben Ard” BC extract, “Currantex 30”) was involved in CCL11 suppression (by 48.55 ± 28.56%); therefore, potentially reducing inflammation [48]. Reduction of inflammation by BC can also be seen in obesity-induced inflammation. The exact mechanism of action needs further investigation, but BCE (~500 mg in humans) has been shown to reduce macrophage infiltration in adipose tissue. Possible reasons for this effect could be enhanced mitochondrial biogenesis, energy expenditure in skeletal muscle, or reduction of the action of inhibitor-κB kinase ε (IKKε) in energy preservation [49].

#### 2.2.4. Dairy Fermented Products with Blackcurrants on the Market

**Figure 4:**
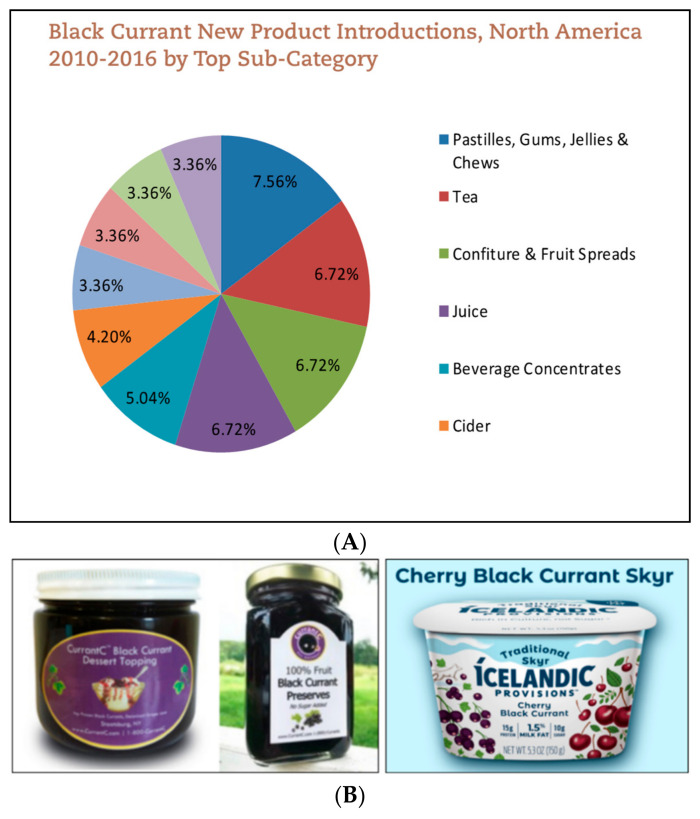
(**A**) Top categories of Blackcurrants products in North America [50]. (**B**) Examples of American made blackcurrants products—dessert toppings, preserves, and Skyr [52,54].

Incorporation of BC in foods has been well established in the European market. In fact, from 2010 to 2016, Europe made up over 50% of the new BC products launched [50]. Worldwide, 3100 new BC products popped up in 2017 alone [50]. BC is also grown in northern Asia and New Zealand [51]. The largest BC product categories include fruit spreads, jams, and beverages [50]. Awareness around BC has increased in North America; of the 3113 new BC products launched in 2017, approximately 4% originated from this region. Similar to Europe, the top product categories include jams, fruit spreads, and beverages [50] (Figure 4A).

Although awareness around BC has increased, BC products are still not widely recognized by the general public. Likely because large brand names have not yet introduced products containing BC in the U.S. Therefore, there are only few dairy-based BC products in the market. The astringent flavor of BC often requires an excess of added sugars to retain the expected sweet flavor when added to yogurt. Some brands resolve this issue with flavor combinations of various berries. For example, Icelandic Provisions launched a Cherry Black Currant Skyr [52]. Currently, the majority of BC products sold in the U.S. are produced by small privately owned businesses. These businesses often grow the BC themselves and advocate for the berries. For example, the founders of CurrantC collaborated with Cornell University Cooperative Extension to reverse the ban on BC [53]. The product line of Currant C includes preserves, concentrates, syrups, and dessert toppings (Figure 4B) [54]. Additionally, CurrantC partners with the International Blackcurrant Association (IBA) to promote new product development using BC [55]. Other U.S. BC products on the market are BC powders at varying anthocyanin levels [55]. Recent health trends would suggest that superfruits like BC are marketable to consumers for their added health benefits. Larger consumer packaged goods (CPG) companies may release products incorporating BC into foods, including yogurt, in the upcoming years.

## 3. Lactic Acid Fermentation to Produce Yogurt

Adding BC to another nutrient-packed food like yogurt is expected to result in synergistic benefits for improving health of consumers. Combining BC and yogurt can provide probiotics, prebiotics, protein, fatty acids, vitamins, and minerals, which contribute to a healthy, disease-preventing snack [56]. According to Saxelin [57], yogurt-type drinks are the fastest growing product category in Europe due to their health impact on supporting the gut microbiota. Lactic acid fermented berries can be added to products like yogurt and also have the potential to be incorporated into other products like a probiotic fruit smoothie, kefirs, ice creams, and probiotic fruit juices [57].

According to the Food and Drug Administration (FDA), to be considered yogurt, dairy ingredients must be cultured with *Lactobacillus bulgaricus* and *Streptococcus thermophilus*. Yogurt cannot contain less than 3.25% milkfat, less than 8.25% milk solids that are not fat, or have a titratable acidity that is less than 0.9%. It must also be pasteurized or ultra-pasteurized before the addition of the bacterial culture. Flavors, colors, stabilizers, and vitamins are optional [58]. In general, a minimum of 10^6^ CFU/mL or gram should be in a probiotic product and 10^8^ to 10^9^ probiotic microorganisms need to be consumed daily by the consumer to have an effect on the body [59].

### 3.1. Chemistry of Yogurt Fermentation

The first step in the yogurt manufacturing process is to homogenize the milk. This causes the fat globules to become smaller and prevents separation of fat and whey during fermentation or storage. Pasteurization then occurs and the milk is heated in order to kill unwanted microorganisms and remove dissolved oxygen to promote the growth of the starter culture. The next step is to cool the milk to around 40–45 °C for optimal growth conditions of the starter culture [60]. *Lactobacillus delbrueckii* subsp. *bulgaricus* and *Streptococcus* subsp. *thermophilus* are the required starter cultures used in the fermentation of yogurt [58]. During incubation, the lactose in the milk is broken down into glucose and galactose by lactase, and these simple sugars are then ingested and metabolized by the bacteria, releasing lactic acid as a waste product [61]. The lactic acid reduces the pH of milk, which causes the casein to precipitate at a pH of 4.6–4.7 [62]. This precipitation occurs because the isoelectric point of casein is 4.6. At this point, there is a decrease in negative charges on casein causing more plus-minus charge interactions. The yogurt is then cooled to stop fermentation, and fruit or flavors are added if desired [60].

Many factors influence yogurt production such as heat, pH, fat content, and the bacteria used. LAB can produce compounds such as bacteriocins, carbon dioxide, hydrogen peroxide, diacetyl, and organic acids such as lactic acid. These compounds can inhibit harmful microorganisms and therefore can extend the shelf life of the product without the addition of preservatives or thermal treatments [63]. Heat treatment of milk affects the consistency and texture of the final yogurt product. As milk is subjected to temperatures of 70 °C or above, most of whey proteins denature and either solubilize or bind casein micelles. The starting pH of the milk affects the proportion of denatured whey proteins that associate with casein micelles and remain soluble. As pH decreases, a larger proportion of whey proteins associate with casein micelles, and as the pH increases a larger proportion of whey proteins remain soluble. In order to produce favorable gel stiffness, an optimal ratio of soluble whey to casein bound whey is needed. The ratio produced by the use of natural milk that did not receive a pH adjustment (roughly pH 6.7) results in yogurt with higher gel stiffness as compared to a pH of 6.2 and 7.2 [64].

After examining the effects of fat content and pre-heat treatment on yogurt, it has been shown that viscosity increased ~100% as the fat content rose from 0.2% to 3% (at the incubation time with the maximum viscosity). In addition, viscosity increased ~57% when the preheat temperature increased from 90 °C to 137 °C (at the incubation time with the maximum viscosity). Two possible mechanisms by which the preheat treatment affects viscosity are an increased number of intermolecular disulfide bridges that are a product of higher temperature, as well as increased water binding capacity that results from whey protein denaturation; both proposed mechanisms result in a more viscous final product [65].

Along with the traditional starter culture consisting of the bacteria *Lactobacillus delbrueckii* subsp. *bulgaricus* and *Streptococcus* subsp. *thermophilus,* adjunct bacterial cultures can provide yogurt with greater quality and depth of flavor and aromaticity. The addition of two different *Lactobacillus plantarum* strains 1-33 and 1-34, in coordination with the starter cultures, resulted in the increase of metabolites that contributed to the flavor of the yogurt, such as acetaldehyde, diacetyl, and acetoin [66]. *L. plantarum* has the potential to enhance the production of novel fermented milk by means of probiotic benefits and enhancement of *Streptococcus thermophilus* growth. The mechanism by which the *L. plantarum* facilitates growth of *S. thermophilus* is unknown [67].

In order to attain amino acids and nitrogen, bacteria contain proteases that can break down peptides in the surrounding yogurt environment. The level of proteolytic activity of starter cultures used to produce yogurt influence the sensory experience of the consumer. Yogurt made with strains that have low to medium proteolytic activity are deemed more favorable than those with strains of high proteolytic activity [68]. Depending on the size of peptides and different fermentation times, the antioxidant activity increased [69]. It is hypothesized that excessive proteolytic activity can contribute to the presence of bitter peptides in the final product. Moreover, excessive hydrolysis of casein may result in a yogurt that is too soft [68].

### 3.2. Yogurt Consumption

Consumers of yogurt benefit from improved metabolic health and body composition as compared to non-consumers [70]. Yogurt is a rich source of vitamin D, potassium, and calcium, as well as protein. Data from the National Health and Nutrition Examination Survey concluded that the consumption of yogurt is associated with increased levels of vitamin D, calcium, and protein [64]; moreover, yogurt consumption is also associated with lower body fat [71]. It is suggested that protein can play a role in weight management by means of appetite control, as this macronutrient is associated with greater satiety. Calcium in the diet promoted an increase in thermogenesis, fatty acid oxidation, lipolysis, and fecal fat excretion. It also caused a decrease in lipogenesis and hunger. All of these factors are reasons calcium is important in relation to preventing obesity [71]. The possible mechanisms for the role of vitamin D in preventing obesity are anti-inflammatory and proapoptotic effects in adipocytes [71]. *Streptococcus thermophilus* can synthesize folates, which facilitates the production of folate-enriched dairy products. When compared to the unfermented counterparts, the fermented dairy products produced with *S. thermophilus* results in a one to six-fold increase in the amount of folate present. The rapid decrease in pH increases folate after fermentation [72].

Yogurt consumption reduces symptoms of lactose intolerance. The probiotic effect of lactose metabolism in humans demonstrated that regular consumption of yogurt increases the activity of β-galactosidase as a result of bacteria colonization in the gut. Fecal samples, from 30 healthy subjects who regularly consumed yogurt, were compared to samples from 21 healthy subjects who did not consume yogurt [73]. This comparative analysis showed that the percentage of samples containing *Lactobacillus bulgaricus* was significantly higher in the group that consumed yogurt (73% in consumers vs 28% in nonconsumers, *p* = 0.003) [73]. Moreover, β-galactosidase activity was higher in this group (85.43 ± 2.51 μmol/min per g protein for consumers compared to 68.90 ± 2.43 μmol/min per g protein for non-consumers, *p* = 0.048). Since lactic acid bacteria (LAB) produce β-galactosidase, it may facilitate digestion of dairy products in those with lactose intolerance [73].

In addition, yogurt contains probiotics, which are live microorganisms that provide the host with health benefits [74]. Probiotics in yogurt may help in the management and prevention of type 2 diabetes (T2D). A group of 44 diabetic adults were divided into two groups in a randomized, double blind manner; one group received 300 g/day of probiotic yogurt while the other group received the same amount of conventional yogurt for an 8-week intervention period. The presence of inflammatory cytokines, such as TNF- and HbA1c levels, were significantly reduced in the experimental group that consumed probiotic yogurt (from 4.36 ± 1.90 to 2.92 ± 1.16 pg/mL, *p* = 0.04, and from 8.24 ± 1.68 to 7.09 ± 1.25%, *p* = 0.032, respectively) [75]. HbA1c levels may also be lowered as a result of the presence of LAB in the gut. These bacteria may have metabolized some of the glucose, decreasing the amount of glucose available for absorption. Moreover, the decreased levels of inflammatory cytokines, such as TNF-α, may be the result of inhibition by LAB in the gut [75].

In summary, the properties of yogurt products can be significantly affected by the strains of bacteria used for fermentation as well as by the physical and chemical environment in which the product is fermented. For example, there is evidence that certain bacteria partake in a symbiotic relationship, such as *L. plantarum* enhancing the growth of *S. thermophilus* in fermented milk products. Furthermore, different strains of bacteria exhibit different metabolic properties, such as different proteolytic activities of *S. thermophilus* and *L. bulgaricus* strains that affect physical and sensory properties of yogurt.

### 3.3. Addition of Other Bacteria

*Lactobacillus plantarum* and *Lactobacillus fermentum* have been tested with milk or yogurt as carriers since these two strains effectively survive gastrointestinal transit [76]. These strains provided inhibition of pathogenic microbial strains (*Listeria monocytogenes*, *Salmonella enteritidis*, and *Escherichia coli* O157:H7). Both bacteria also overproduced riboflavin, making them viable options for yogurt products with increased amounts of this vitamin [76].

Furthermore, there is evidence that probiotic yogurt containing *Lactobacillus acidophilus* La5 and *Bifidobacterium lactis* Bb12, positively affected metabolic factors in individuals with nonalcoholic fatty liver disease (NAFLD) [77]. Subjects in the experimental group with NAFLD (*n* = 36) consumed 300 g per day of probiotic yogurt containing *Lactobacillus acidophilus* La5 and *Bifidobacterium lactis* Bb12. The control group (*n* = 36) with NAFLD consumed conventional yogurt. The experimental group showed significant improvement in metabolic markers such as serum levels of alanine aminotransferase, aspartate aminotransferase, total cholesterol, and low-density lipoprotein (LDL). One proposed mechanism for LDL reduction in these patients was the assimilation and binding of the LAB to dietary cholesterol, which would decrease the amount of cholesterol available for intestinal absorption. Another hypothesis involves the action of sphingolipids in yogurt and the cell membranes of bacteria that can transport and metabolize dietary cholesterol [77].

Probiotic yogurt with *Lactobacillus acidophilus* La-5 and *Bifidobacterium* BB-12 may improve intestinal function in patients with irritable bowel disease (IBD) in comparison to a group without IBD that consumed probiotic yogurt [78]. The 8-week intervention revealed that the levels of *Lactobacillus* and *Bifidobacterium* were significantly higher in the stool of the experimental group that received the treatment as compared to the group that received placebo from 6.1 ± 0.4 to 8.3 ± 0.4 CFU/g, (*p* < 0.001) and from 7.3 ± 0.3 to 10.5 ± 0.5 CFU/g (*p* < 0.001), respectively. The results suggested that the introduction of such bacteria in the gastrointestinal tract of patients with IBD may assist in intestinal function and overall management of the disease [78].

There is evidence that the use of the novel bacterial strain, *Lactobacillus mucosae,* in the production of yogurt results in increased antioxidant activity in vivo [79]. Researchers procured bacterial isolates from the gastrointestinal tracts of Gaotian villagers in China. Three strains of procured bacteria (*Lactobacillus mucosae* and two strains of *Lactobacillus plantarum*) were used to prepare yogurt which were then fed to experimental groups of aging mice; the control group was fed vitamin C. Of the three experimental groups, the one that consumed the yogurt prepared using *L. mucosae* showed the greatest antioxidant activity (>80%). The inclusion of this bacterial strain in probiotic yogurt may enhance antioxidant capacity linked to consumer benefits [79].

*L. plantarum* YS5 exhibited hypocholesterolemic activity in vivo, making it a viable option in the production of health promoting foods. Male rats were fed high-fat diets and split into two experimental groups, one of which received an *L. plantarum* YS5 supplement while the other did not. After the 8-week intervention period, the group that received the supplementation (10^6^–10^7^ CFU/g) showed decreased serum total cholesterol, triglycerides, and LDL cholesterol levels, while also showed increased high-density lipoprotein (HDL). Moreover, *Lactobacillus plantarum* YS5 strain exhibited favorable resistance to acid and bile conditions, antagonistic activity against bacterial pathogens, hydrophobicity, and autoaggregation. *L. plantarum* YS5 strain showed great potential as a health-promoting probiotic used in the production of yogurt [80].

In summary, yogurt is a good source of vitamin D, potassium, calcium, and protein, and shows potential antiobesity properties. In patients with T2D, probiotic yogurt consumption was correlated with significant decreased levels of inflammatory cytokines, decreased levels of HbA1c, and LDL cholesterol. Similar results were observed in patients with non-alcoholic fatty liver disease. In patients with IBD, a significant increase in intestinal bacteria may help in disease management. Certain strains of bacteria can confer health benefits in yogurt, such as the ability of *L. mucosae* to increase antioxidant activity and *L. plantarum* YS5’s, cholesterol lowering activity. In addition, certain bacteria have unique nutrient synthesizing activity, such as the folate synthesis of *S. thermophilus* as well as riboflavin synthesis of *L. plantarum* and *L. fermentum* that can be promoted for manufacturing vitamin-enriched yogurt.

## 4. Blackcurrants Fermented with Lactic Acid Bacteria

### 4.1. Effect of Fermentation on Chemical Composition and Antioxidant Capacity of Blackcurrants

**Figure 5:**
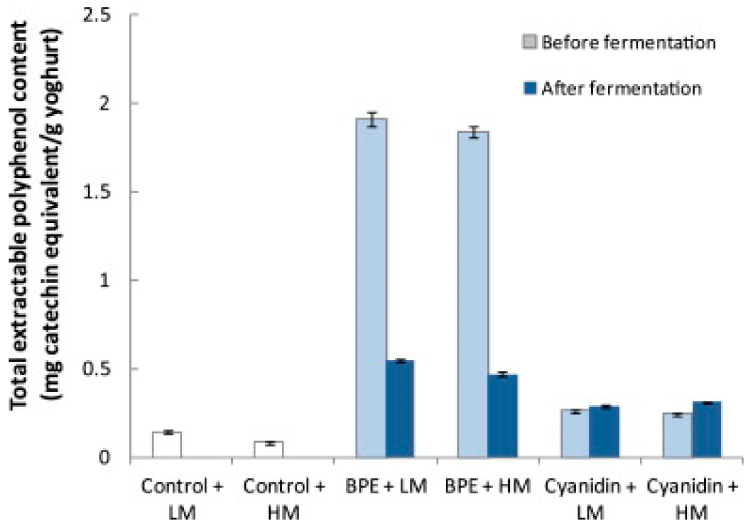
Total extractable polyphenol content of the control and polyphenol-enhanced yogurt when Blackcurrants Polyphenol Extract (BPE) was added before and after fermentation. The standard deviation of the mean is presented with error bars. Abbreviations: Low methoxyl pectins (LM) high methoxyl pectins (HM), blackcurrant extract (BCE), cyanidin 3-*O*-β-glucopyranoside chloride (cyanidin) [82]. Elsevier Copyright Clearance Center’s RightsLink^®^ service.

Fermentation of foods may increase the extent to which polyphenols become available for use in biological processes [81]. The development of a BC yogurt can be approached by two experimental procedures. Either the BC can be fermented independently and then added to the fermented yogurt, or the BC can be fermented along with the yogurt. A study assessing the effect of adding BCE to yogurt pre and postfermentation, found that the total extractable polyphenol content was 3.5–3.9 times larger when BCE was added pre-fermentation (Figure 5) [82]. The fermentative activities impacted the polyphenol makeup of the drinkable yogurt, indicating that the polyphenols were transformed into more bioavailable or stable forms by the fermentation processes. More research into the specific catabolic mechanisms of polyphenol breakdown should be conducted to further understand how fermentation affects polyphenols. It is important to note the BCE added postfermentation was not independently fermented, indicating the only variation between the two samples was the fermentation of BC [82].

Another study found that adding BC prior to fermentation resulted in small phenolic acids in the yogurt product. These phenolic compounds have antioxidant activity and can contribute to scavenging of radicals. Such phenolic molecules were not present when the BCE was included post fermentation [82], suggesting that fermentation breaks larger compounds down into phenolic acids [74]. It was also found that fermentation of mixed berry juice caused a rise in the antioxidant activity, from 209.57 ± 2.93 to 268.30 ± 1.75 µm TE/g [83]. In addition to the mixed berry juice, acai berry juice and blackberry juice showed a 31% and 16% increase in antioxidant capacity per gram of berry from LAB fermentation. Specifically, the antioxidant profile of the acai berry was much greater than the other berry profiles examined [84]. To our knowledge, there has been little research focused on the antioxidant capacity of BC before and after fermentation. However, existing studies suggest that BC could perform just as well as other more studied berries due to their high polyphenol content.

**Figure 6:**
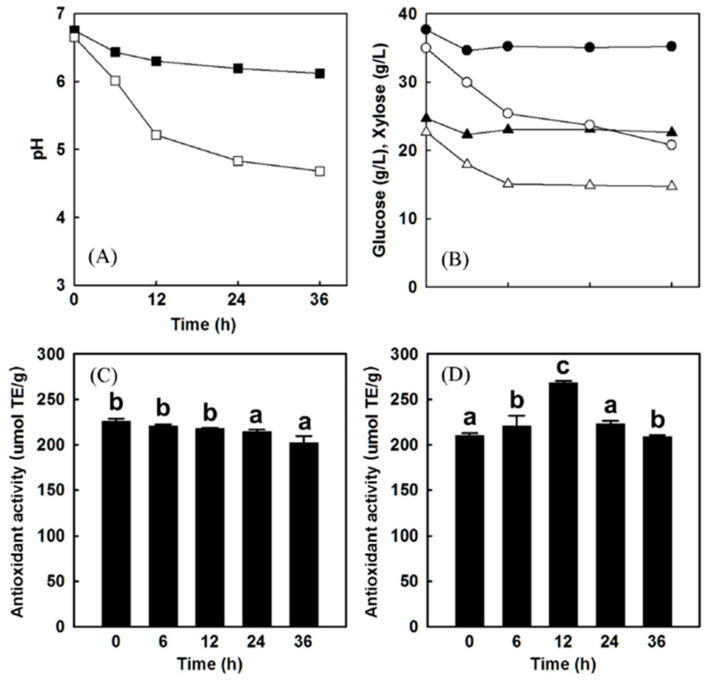
Results after *L. plantarum* LP-115 fermentation of the mixed berry juice (73% acai, 17% aronia, 10% cranberry) (**A**) Change in pH over time with (open square) and without (closed square) L. plantarum LP-115 inoculation. (**B**) Amount of glucose (circles) and xylose (triangles) consumptions over time with (open symbols) or without (closed symbols) addition of L. plantarum LP-115. (**C**) Change in antioxidant activities over time without inoculation of *L. plantarum* LP-115. (**D**) Changes in antioxidant activities over time with inoculation of *L. plantarum* LP-115. Error bars indicate standard deviation. Values with the same letter are not statistically significant based on Scheffe’s multiple range test [83]. Provided by the Springer Nature SharedIt content-sharing initiative https://rdcu.be/b92Xd.

The fermentation of mixed berry juice (Figure 6), acai berry juice, and myrtle berries with *L. plantarum* increased the antioxidant activity postfermentation [83,84,85]. *L. plantarum* LP-115 caused a 20–30% rise in the antioxidant activity of the fermented acai berry juice [84] and increased the antioxidant activity of the myrtle berries compared to the control [85]. Evidence has shown that certain *L. plantarum* species contain tannase (tannin acyl hydrolase), an enzyme that hydrolyzes ester bonds of hydrolysable tannins (not condensed tannins) producing phenolic compounds gallic acid with antioxidant properties [86,87]. When the berries are fermented separately and then added to the yogurt, *L. plantarum* may be effective in increasing the polyphenol content. In addition, the tannase activity in certain *L. plantarum* species decrease astringency of the berries by hydrolyzing tannins [87]. However, from what is currently known, BC contain mostly condensed tannins (flavan-3-ol) and not hydrolysable tannins (gallitannins and ellagitannins) like other berries such as blackberries, strawberries, and raspberries [88]. Therefore, tannase activity most likely will not have much effect on BC antioxidant levels. Park et al. [83] suggested that hydrolase or other metabolites from bacteria may improve antioxidant activity of different fruits during fermentation. In addition, if the fruit is added prefermentation, *S. thermophiles* and *L. bulgaricus* must be present, to be considered a yogurt. Further research is required on the impact of using the three cultures together on antioxidant capacity and yogurt functionality.

LAB fermentation increased antioxidant capacity for other foods such as jujube juice and apple juice. For the jujube juice, antioxidant capacity was positively correlated with caffeic acid and rutin concentrations [89]. For the apple juice, the antioxidant capacity was also related to the increase in caffeic acid and phlorizin concentrations after fermentation [90]. LAB fermentation caused a bioconversion of isoflavone glucosides to aglycones, and the rise in antioxidant scavenging capacity was correlated with the isoflavone aglycone concentration [91].

### 4.2. Important Factors That Influence the Blackcurrant Yogurt Product

A dairy application for a BC-fermented product is ideal due the high protein content of dairy, which may help to mask the astringency of BC, improve taste, and minimize the use of sugar as ingredient. Milk proteins are believed to protect polyphenols by forming protein–polyphenol complexes. Casein and whey proteins bind to polyphenols by hydrophobic and hydrogen bonding [92]. Whey proteins, β-lactoglobulin (β-LG), and a β-LG with caseinomacropeptides (CMP) mixture reduced astringency of wine. It was suggested that the mechanism of action was that the β-LG interacted mainly via hydrophobic interactions and hydrogen bonding with tannins that led to the hydrophobic areas of the proteins being covered by tannins followed by aggregation and precipitation [93].

*Streptococcus thermophiles* and *L. delbrueckii* subsp. *bulgaricus* have long been used in yogurt due to their harmonious relationship with changes in pH [82]. *S. thermophiles* actively ferments lactic acid until the pH reaches 5.0, where the acidic environment diminishes the activity of *S. thermophiles* and stimulates *L. bulgaricus* [94]. The addition of polyphenols before fermentation, through BCE, caused significant growth of both *S. thermophiles* and *L. bulgaricus* [82]. Thus, it can be predicted that BCE may promote the activity of these two cultures. To our knowledge, there has been little research conducted on how *L. plantarum* interacts with *S. thermophiles* and *L. bulgaricus* throughout yogurt fermentation. *L. plantarum* has been found to adapt well to various environments and have high metabolic flexibility [95].

Berries naturally contain lactic and acetic acid bacteria as well as yeasts. Therefore, natural fermentation from these microorganisms is possible [96]. The bacteria used for the fermentation can either come from the raw material itself, also known as spontaneous fermentation, or it can come from the use of a starter culture. It is important to consider that spontaneous fermentation can have some risks such as the presence of microbial pathogens and toxic byproducts such as mycotoxins, ethyl carbamate, and biogenic amines that can be a safety risk for the consumer. The use of starter culture technology has allowed fermentations to be safer and more desirable since different biological activities can affect the fermentation. For example, starter cultures can have faster acidification activity, reduction of fermentation time, reduction of toxic compounds, and affect the palatability and aroma. More research is needed to determine the optimum starter culture to use to ferment BC, so the bacteria survive until the use-by date of the product and the desired health benefits and taste are obtained [97].

It is important to consider the optimum growth conditions of the LAB fermenting the product. For example, *L. plantarum* strains did not grow below pH 3 but most strains grow around pH 4.5 to 6.5 [98]. BC have a pH around 2.7–3.0 [24]; therefore, the pH of the juice would need to be increased with a more basic ingredient to allow the bacteria to grow if the BC juice is fermented on its own and not with milk. The addition of nutrients such as soy-peptone, glucose, yeast extract, or magnesium sulfate can help to maximize the growth of the bacteria [99].

### 4.3. Health Benefits of Fermented Blackcurrants Products in Dairy

Along with the probiotic effect from the use of live microorganisms that promote gastrointestinal health, fermented foods can enhance the health promoting properties of foods by transforming substrates to bioactive compounds as end products [100]. Prebiotics found in the fruit can contribute in supporting probiotic bacteria in both the large intestine and yogurt itself [101]. One study found that BC pomace yogurt has antidiabetic properties by inhibiting α-amylase (~60%), α-glucosidase (~90%), and DPP-IV (~60%) enzymes after 28 days of storage at 4 °C. This may be due to the peptides released from the caseins in the yogurt during fermentation and phenolic compound interaction with milk proteins. It is important to note that this study utilized pomace of BC (the skins and seeds) and not the juice. Therefore, more research is needed on antidiabetic effects of yogurt with BC juice, especially on humans [102]. In addition, a dietary pattern of fruit and dairy has been related with a decreased risk of metabolic syndrome in Koreans [103]. It suggested that a combination of several foods had a larger impact than individual food components on health. Several synchronous predictors of cardiovascular disease and adult-onset diabetes define the metabolic syndrome. These characteristic symptoms include insulin resistance, obesity, lowered HDL, and elevated triglycerides and LDL [104]. The predicted increase postfermentation of polyphenols and antioxidant capacity would strengthen the health benefits of a BC yogurt product. Studies have shown antioxidant-rich foods may prevent diseases such as cancer, cardiovascular diseases, T2D, and Parkinson’s disease [105].

## 5. Conclusions

BC yogurt possesses not only prebiotics and probiotic benefits, but also contains several bioactive compounds (phenolics) that can prevent certain disease conditions such as metabolic syndrome, cardiovascular diseases, IBD, neurological diseases, and T2D. Fermenting berries, in general with LAB, can increase antioxidant capacity, but there is limited evidence of this for BC. Along with its health benefits, fermentation of BC in yogurt may improve the astringent taste of the berry due to the addition of protein and change in pH. However, more human intervention studies are needed on the health benefits and sensory aspects of BC fermented with LAB.

## Figures and Tables

**Table 1:** Phenolic compounds present in 21 blackcurrant cultivars grown in five different countries in Europe.

Compound#	Tentative Identification	Abbreviation
**Anthocyanins**		
1	**delphinidin 3-*O*-glucoside**	De-Glu
2	**delphinidin 3-*O*-rutinoside**	De-Rut
3	**cyaniding 3-*O*-glucoside**	Cy-Glu
4	**cyaniding 3-*O*-rutinoside**	Cy-Rut
5	petunidin 3-*O*-glucoside	Pt-Glu
6	petunidin 3-*O*-rutinoside	Pt-Rut
7	cyaniding 3-*O*-arabinoside	Cy-Ara
8	pelargonidin 3-*O*-glucoside	Pl-Glu
9	pelargonidin 3-*O*-rutinoside	Pl-Rut
10	peonidin 3-*O*-glucoside	Po-Glu
11	peonidin 3-*O*-rutinoside	Po-Rut
12	malvidin 3-*O*-glucoside	Ma-Glu
13	malvidin 3-*O*-rutinoside	Ma-Rut
14	delphinidin 3-*O*-(6″-coumaroyl)-glucoside	De-coGlu
15	cyaniding 3-*O*-(6″-coumaroyl)-glucoside	Cy-coGlu
**Flavonols**		
16	**myricetin 3-*O*-rutinoside**	My-Rut
17	**myricetin 3-*O*-galactoside**	My-Gal
18	**myricetin 3-*O*-glucoside**	My-Glu
19	**myricetin 3-*O*-arabinoside**	My-Ara
20	**myricetin 3-*O*-(6″-malonyl)-galactoside**	My-maGal
21	quercetin 3-*O*-rutinoside	Qu-Rut
22	quercetin 3-*O*-galactoside	Qu-Gal
23	quercetin 3-*O*-glucoside	Qu-Glu
24	quercetin 3-*O*-arabinoside	Qu-Ara
25	quercetin 3-*O*-(6″-malonyl)-glucoside	Qu-maGlu
26	kaempferol 3-*O*-rutinoside	Ka-Rut
27	kaempferol 3-*O*-galactoside	Ka-Gal
28	isorhamnetin 3-*O*-glucoside	Is-Glu
29	**myricetin aglycone**	Myagly
30	kaempferol 3-*O*-(6″-malonyl)-glucoside	Ka-maGlu
31	isorhamnetin 3-*O*-(6″-malonyl)-galactoside	Is-maGal
32	**myricetin-hexoside-deoxyhexoside**	My-hex-deox
33	isorhamnetin 3-*O*-(6″-malonyl)-glucoside	Is-maGlu
34	quercetin aglycone	Quagly
**Phenolic Acid Derivatives**		
35	5-*O*-caffeoylquinicacid	5-CaQA
36	4-*O*-caffeoylglucose	4-Ca-Glu
37	1-*O*-caffeoylglucose	1-Ca-Glu
38	**coumaroylquinic acid isomer**	CoQA
39	**3-*O*-coumaroylquinicacid**	3-CoQA
40	**4-*O*-coumaroylglucose**	4-Co-Glu
41	**1-*O*-coumaroylglucose**	1-Co-Glu
42	3-*O*-caffeoylquinicacid	3-CaQA
43	feruloylglucose isomer	Fe-Glu
44	1-*O*-feruloylglucose	1-Fe-Glu
45	(*E*)-caffeoyloxymethylene-glucopyranosyloxy-(*Z*)-butenenitrile	Ca-meGlu-B
46	(*E*)-coumaroyloxymethylene-glucopyranosyloxy-(*Z*)-butenenitrile	Co-meGlu-B1
47	(*Z*)-coumaroyloxymethylene-glucopyranosyloxy-(*Z*)-butenenitrile	Co-meGlu-B2
48	(*E*)-feruloyloxymethylene-glucopyranosyloxy-(*Z*)-butenenitrile	Fe-meGlu-B
**Flavan-3-ols**		
49	gallocatechin	GCat
50	epigallocatechin	EGCat
51	(+)-catechin	Cat
52	(−)-epicatechin	ECat
**Other Phenolics**		
53	aureusidin glucoside	Au-Glu
**Organic Acids**		
54	malic acid	MaA
55	**citric acid**	CiA
56	quinic acid	QuA
57	ascorbic acid	AsA
**Sugars**		
58–60	**fructose anomers**	Fru
61,62	glucose anomers	Glu
63	sucrose	Sur

Compounds with the highest concentration are bolded. Compounds were identified using ultra-performance liquid chromatography-diode array detector electrospray-ionization tandem mass spectrometry (UPLC-DAD-ESI_MS). Source: adapted from [12].

**Table 2:** Antioxidant capacity (μmol of Fe^2+^/g) and total content of vitamin C, anthocyanins, and flavonols (nmol/g of fresh weight) contributing to antioxidant capacity of blackcurrants, blueberries, raspberries, red currants, and cranberries.

Compound	Blackcurrant	Blueberry	Raspberry	Red Currant	Cranberry
Vitamin C	2328 (18)	115 (0)	1014 (11)	313 (47)	1107 (23)
Anthocyanins	5521 (73)	4810 (84)	885 (16)	328 (21)	725 (39)
Flavanols	514 (5)	751 (14)	67 (0)	69 (4)	456 (10)
Antioxidant capacity FRAP (μmol of Fe^2+^/g)	51.6 ± 1.2	30.0 ± 1.9	27.7 ± 1.1	24.6 ± 0.5	18.6 ± 0.3

Numbers in parentheses are percentages of the total antioxidant capacity. Source: modified from [19].

**Table 3:** Health benefits of blackcurrants based on human studies.

Health Benefit	Model Used	Results	Reference
Treat neurological conditions such as PD	Plasma and CSF collected from 11 male patients before and after 300 mg of BCA was taken twice daily for 4 weeks	Significant increase in cGP with an increase in BCA dose (*p* < 0.01). Strong correlation between the concentration of cGP in CSF and plasma (*p* = 0.01).	[29]
Neuroendocrinological and Cognitive benefits	In a randomized, double-blind, placebo-controlled, cross over study, 36 healthy adults from Auckland NZ aged 18 to 35 were given either 0 mg of PP (Control) or 525 ± 5 mg of polyphenols per 60 kg of bodyweight from ANC-enriched BC extract or 142 mL of BC fruit juice	Significant inhibition of MAO-A and MAO-B. Amount of processing and BC cultivar is a major factor in neuroendocrinological and cognitive benefits.	[32]
Treat neurological conditions	In a double-blind, placebo-controlled, randomized cross- over study, 8 healthy male aged 20–35 consumed NZ Blackadder juice. Measurements were obtained at baseline 15, 30, 45, 60, 100, 120, 150, 180, 240 min, and 24 h post dose	A fast, absolute, and reversible inhibition of blood platelet MAO-B (*p* < 0.001). Significant but delayed reduction in plasma prolactin (*p* < 0.001) following the consumption of BC juice.	[33]
Positive affective responses during a self-motivated exercise	In a parallel, randomized controlled study, 40 healthy sedentary male and female participants drank a BC juice (4.8 mg/kg bodyweight) or PLA. After 3- or 5-min intervals on a treadmill, heart rate and exertion (ES) or feeling/mood scores (FS) were measured. Markers were measured pre- and postexercise	A 90% decline in platelet MAO-B activity. ES increased in both groups; however, ES for the BC group were lower than the PLA group (*p* < 0.05). FS was inversely related to ES only for the PLA group (r^2^ = 0.99, *p* = 0.001).	[34]
Mood and Attention	In a randomized, double-blind and placebo-controlled crossover design, 9 healthy adults consumed either BC juice (500 mg polyphenols/drink) or PLA. Neuronal activity was determined related to cognitive performance using EEG.	Reduced anxiety, greater alertness, lower fatigue and small increase in reaction times was seen after the consumption of a single serve BC juice.	[35]
Exercise Recovery	Healthy adults (32) who exercised daily, rowed for 30 min prior to consumption of BC extract (PLA, 0.8, 1.6, or 3.2, mg/kg total ANC). Blood samples were taken throughout	Post-exercise HR was significantly (*p* < 0.05) lower in participants who had consumed 0.8 or 1.6 mg/kg BAE compared to the PLA. Consumption of 1.6 or 3.2 mg/kg BAE showed elevated pre-exercise plasma oxidative capability and lowered post-exercise plasma carbonyl levels compared to PLA. Neutrophil phagocytic capability was preserved after consumption of BAE before exercise	[36]
Exercise recovery	In 2 double-blind placebo-controlled trials, 18 healthy adults with moderate daily physical activity, consumed BAE (3.2 mg/kg ANC) or PLA 1 h prior to 30 min of rowing exercise for 5 weeks	Significant reduction in lipid oxidation product MDA (*p* < 0.05). BAE promotes beneficial, protective antioxidant/anti-inflammatory cellular events.	[37]
Improve cardiovascular function	Fifteen endurance male cyclists were randomly assigned to consume different amounts of BCE for 7 days with a 14-day washout (300, 600, or 900 mg/day). Results for cardiovascular function during supine rest were collected.	Cardiac output and stroke volume increased, and peripheral resistance decreased compared to the control.	[38]
Improved Athletic Performance and Recovery	In a double blind, placebo controlled, randomized design, 13 triathletes with >3 years of experience consumed 6 g of BC powder for 7 days and physiological and cardiovascular responses were obtained.	After intake of BC cycling intensity was 6% higher, and there was no effect on heart rate and oxygen uptake. When resting, stroke volume (25%) and cardiac output increased (26%). Total peripheral resistance decreased (16%) and blood pressure and heart rate were not affected.	[39]
Improved Athletic Performance	In a double blind, randomized, crossover design, 14 healthy men consumed BCE (300 mg/day with 105 mg of ANC) or a placebo. After 30 min of cycling and a 16.1 km time-trial, results were collected.	With BCE consumption, fat oxidation increased by 27% and the 16.1 km performance was improved (2.4%). Plasma lactate was higher after the 16.1 km time-trial when BCE was consumed.	[40]
Improved Athletic Performance and Recovery	In a double-blind, randomized, crossover design, 13 active males consumed BCE (300 mg/day with 105 mg of ANC) or a placebo for 7 days and completed a series of sprints at different speeds with 15 s of low intensity running and 1 min of rest between sprints.	BCE consumption increased total running distance by 10.6% compared to the placebo. Blood lactate was higher at exhaustion for those who consumed BCE. Greater lactate change after 15 min of recovery.	[41]
Improved Athletic Performance	In a double-blind, randomized, crossover design, 18 male climbers consumed BCE supplementation (600 mg/day with 210 mg ANC) or placebo for 7 days. Climbing performance was assessed after 3 climbs.	Pull-up performance was not affected Heart rate and total climbing time increased compared to the placebo Fatigue was present regardless of supplementation or not	[42]
Diabetes	In a randomized, double-blind, crossover trial, 14 men and 9 post-menopausal women consumed a BC extract drink with 150, 300, or 600 mg of total ANC before a high-carbohydrate meal or the PLA. Plasma glucose, insulin, GIP, GLP-1, Plasma 8-isoprostane F_2α_, and arterial stiffness was measured at 0 and 120 min.	Plasma glucose, plasma insulin, plasma GIP, and plasma GLP-1 concentrations were significantly reduced compared to the control.	[44]
Reduce acute endothelial dysfunction caused by smoking	In a randomized, double-blind trial on young, healthy male smokers (13) and nonsmokers (11), the effects of FMD and skin temperature were tested after no supplement, 50 mg BCA, or 50 mg BCA plus Vit E.	After consumption of 50 mg BCA, FMD decreased 1h after smoking and restored to the baseline level 2h after smoking. The skin temperature decreased in the smoker group after smoking compared with that in the nonsmoker group, whereas after BCA supplements, it was higher in the smoker group compared to nonsmoker group.	[45]
Reduce arterial stiffness and blood pressure	In a randomized, double-blind, placebo-controlled, crossover design study, fourteen older adults aged 73.3 ± 1.7 years took either a 7-day course of placebo or two capsules of NZBC extract (each 300 mg capsule contains 35% BC extract) followed by a washout period of 28 days and then switched trials after. Carotid-femoral pulse-wave velocity and central blood pressure were measured.	Carotid-femoral pulse-wave velocity and central blood pressure decreased from both the baseline and placebo. No effects were observed on serum lipids.	[47]

ANC—anthocyanin, BAE—blackcurrants anthocyanin-rich extract, BC—Blackcurrants, BCA—Blackcurrants anthocyanin, cGP—cyclic glycine-proline, CSF—cerebrospinal fluid, EEG—electroencephalography, FMD—flow-mediated dilatation, GIP—glucose-dependent insulinotropic polypeptide, GLP-1—glucagon-like peptide, MAO—monoamine oxidase, MDA—malondialdehyde, NZ—New Zealand, PLA—placebo, PP—polyphenol.
